# Mapping AI regulation in health care with the Health & AI Policy Index

**DOI:** 10.1038/s41746-026-02734-y

**Published:** 2026-05-25

**Authors:** Will Moss, Benjamin S. Glicksberg, Sabina Lim, Alexis Zebrowski, Girish N. Nadkarni

**Affiliations:** 1 Windreich Department of Artificial Intelligence and Human Health, Icahn School of Medicine at Mount Sinai, New York, NY USA; 2 Hasso Plattner Institute for Digital Health at Mount Sinai, Icahn School of Medicine at Mount Sinai, New York, NY USA; 3 Jeb E. Brooks School of Public Policy, Cornell University, Ithaca, NY, USA; 4 Government Affairs, Icahn School of Medicine at Mount Sinai, New York, NY USA; 5 Department of Psychiatry, Icahn School of Medicine at Mount Sinai, New York, NY USA; 6 Department of Emergency Medicine, Icahn School of Medicine at Mount Sinai, New York, NY USA; 7 Department of Population Health Science and Policy, Icahn School of Medicine at Mount Sinai, New York, NY USA

**Keywords:** Business and industry, Health care, Medical research, Scientific community, Social sciences

## Abstract

Artificial intelligence (AI) policy for health care is expanding in a fragmented landscape. In a January 1, 2026 snapshot of the Health & AI Policy Index (240 policies), we find that transparency-oriented advisory instruments dominate, equity and safety are usually addressed within broader governance requirements, and obligations fall mainly on providers, regulators, and developers. We describe how this health-specific registry can guide oversight decisions by health systems, vendors, and policymakers.

## Introduction

Artificial intelligence (AI) tools are being introduced into health care at a rapid rate. As pilots and limited deployments expand, questions about safety, fairness, privacy, and accountability are prompting new laws, regulatory actions, guidance, and standards^1,2^. For health-sector and policy stakeholders, however, this emerging health-AI policy space is difficult to navigate: relevant rules are spread across jurisdictions, take different legal forms, and change over time.

**Fig. 1:**
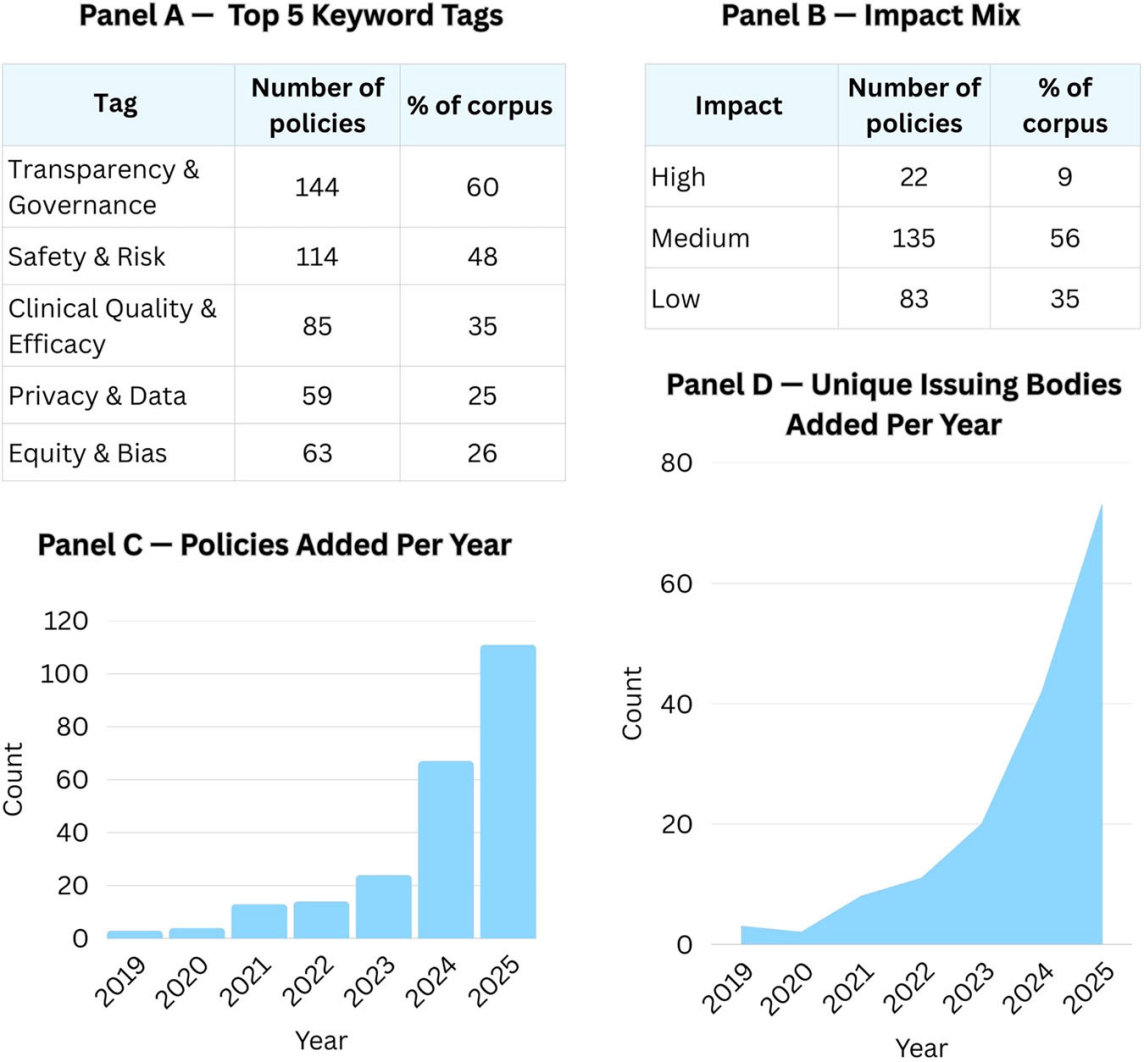
Characteristics of policies in the January 1, 2026, HAPI analytic snapshot (N = 240). **A** Distribution of the five keyword tag families, showing that Transparency and Governance appears on 144 policies (60% of the corpus), followed by safety and risk, clinical quality and efficacy, privacy and data, and equity and bias. **B** Impact mix, with 22 high-impact policies (9%), 135 medium-impact policies (56%), and 83 low-impact policies (35%). **C** Number of policies added per year from 2019–2025, illustrating a sharp increase in 2024–2025. **D** Number of unique issuing bodies added per year over the same period, showing a parallel rise in the diversity of actors entering the health-AI policy space.

In the United States, there is no single omnibus health-AI law and no single regulator responsible for all aspects of oversight. Governance has instead emerged as a patchwork across levels and institutions, with state legislatures, federal agencies, international institutions, professional bodies, and standards organizations each advancing their own AI-related measures. Slow federal legislation and faster state and sectoral experimentation, together with a sharp increase in policy volume and diversity of issuers from 2023 through 2025 (Fig. 1), have further fragmented health-AI governance.

Several tools exist to help track AI-related law and policy, including state AI maps, law-firm resources, and global inventories of national AI strategies and cross-sector statutes (e.g., Ropes and Gray Health AI Atlas and the OECD AI Policy Navigator). However, most focus on a single jurisdiction or instrument type and are not designed for health-sector decision-makers, who lack a comprehensive and health-specific view of policies that shape AI design, deployment, and oversight.

The Health & AI Policy Index (HAPI; https://www.healthaipolicy.org) was developed to help meet that need. HAPI is a public, nonpartisan registry of policies relevant to AI in health care that aggregates U.S. state and federal measures, sector-specific regulations, international frameworks, and voluntary standards into a single structured dataset accessible through an intuitive web interface. Entries are screened for AI and health relevance, tagged for key themes, stakeholders, and impact, and linked to source text, prioritizing official sources where available; users can sort and filter for these tags or use a Trends view that visualizes patterns over time.

In this Perspective, we use a HAPI snapshot frozen on January 1, 2026 to describe the health-AI policy landscape and how the index can support decision-making. We outline how HAPI is built and maintained, use the resulting corpus to characterize recent patterns in health-AI policy activity, and then show how different users can apply the index to navigate a fragmented health-AI governance environment.

## Methods

For this Perspective, we analyzed a frozen snapshot of the Health & AI Policy Index (HAPI) as of January 1, 2026. The analytic dataset includes 240 policies across five modules: U.S. state policies, U.S. federal policies, sector-specific regulations, international frameworks, and voluntary standards. Policies in this snapshot span 2016–2025; Fig. 1 and Supplementary Table 1 summarize annual counts and new issuing bodies from 2019 onward. The public HAPI website continues to be updated as new policies are issued or revised, but subsequent changes do not alter this analytic snapshot, which is preserved separately for reproducibility (Supplementary Data 1).

HAPI focuses on policies that materially affect the development, evaluation, deployment, or oversight of AI in health care. Items are included when they satisfy both AI relevance (such as explicit regulation of artificial intelligence or machine-learning systems) and health relevance (including effects on health care delivery, public health operations, or health-related data, safety, or equity). Policies are excluded when AI is mentioned only in passing, duplicates an existing entry, or is expected to have an insignificant impact on health care.

Policies are identified through regular checks of official sources, screened against inclusion criteria, and entered into the HAPI database. Each record is annotated with a name, module, issuing body, key dates, policy type, and linked to source text, prioritizing official sources where available. Policy type distinguishes binding instruments such as laws and final rules from softer governance tools such as executive orders, guidance, frameworks, standards, resolutions, and commissions. Each policy has a brief summary and health care implications paragraph drafted from the primary text by the HAPI editor, who also makes final decisions about inclusion and how metadata and annotations are coded.

Policies can be assigned up to five keyword tags (Transparency & Governance, Safety & Risk, Clinical Quality & Efficacy, Privacy & Data, Equity & Bias) and five stakeholder tags (Providers & Health Systems, Patients & Public, Payers & Purchasers, Developers & Vendors, Regulators & Government). These tags are deliberately coarse, omitting more granular domains such as liability, reimbursement, or detailed data-provenance requirements, as a trade-off to keep the system usable and visual summaries stable while still helping users quickly spot relevant policies. Definitions and examples for tag assignments are provided in Supplementary Note 1.

Each policy receives a single impact level, high, medium, or low, using a judgment-based rubric that considers legal force, specificity, and expected real-world influence. High-impact policies set concrete expectations health actors are likely to act on; medium-impact instruments shape practice more indirectly or in narrower contexts; and low-impact policies mainly signal intent or prompt discussion with limited near-term operational consequences. Impact levels are intended as an ordinal triage signal rather than a precise measure of legal force or effect, but in practice, high-impact items tend to align with more binding “hard-law” instruments, whereas medium- and low-impact items often reflect softer, advisory measures. Illustrative borderline examples are included in Supplementary Note 1, and to provide a basic check on how consistently the rubric could be applied by others, two independent reviewers with expertise in AI in health care recoded impact levels for 10 percent of policies randomly selected across modules, blinded to the original labels. Simple agreement with the editor’s ratings was 58% and 63%; discrepant cases were resolved by discussion.

To characterize how the most consequential policies operate in practice, we classified each high-impact policy into a single primary governance mechanism. We defined five mutually exclusive mechanism categories, distinct from the topical keyword tags: substantive use conditions and restrictions; organizational governance and processes; information, documentation, and disclosure duties; equity, safety, and assurance regimes; and strategy, capacity-building, and exploratory actions. All high-impact policies in the analytic snapshot were reviewed by the HAPI editor and assigned one primary mechanism based on the full policy text and summary (Supplementary Table 2). Because many policies pursue several aims at once, we did not code a primary-purpose variable and instead use keyword tags and, for high-impact items, primary mechanisms as a pragmatic summary of their main features.

To see whether equity and safety concerns tend to be addressed on their own or embedded within broader transparency and performance requirements, we summarized co-occurrence among keyword tags by counting how often Equity & Bias and Safety & Risk tags appeared together with Transparency & Governance and Clinical Quality & Efficacy tags (Supplementary Table 3). Then, to see which groups policies most directly treat as responsible for or affected by health-AI governance, we summarized stakeholder tag distributions across the snapshot (Supplementary Table 4).

Analyses summarize the composition of the policy mix, the growth and diffusion of activity across issuing bodies, and how equity and safety concerns and stakeholder responsibilities are positioned within this landscape.

## Results

Across the 240 policies in the January 1, 2026 snapshot, we organize the results around three descriptive patterns. First, the current mix is dominated by transparency- and governance-oriented instruments of medium or low impact, with relatively few high-impact policies. Second, policy activity has accelerated in recent years and is now spread across a growing number of issuing bodies (Fig. 1). Third, equity and safety concerns are usually embedded within broader governance and performance requirements, and concrete obligations fall mainly on providers and health systems, regulators, and developers rather than patients or payers.

### Transparency-oriented policies predominate

Across the corpus, Transparency & Governance is the most frequent keyword tag, appearing on 144 policies (60%) (Fig. 1A). Safety & Risk appears on 114 policies (48%), followed by Clinical Quality & Efficacy (85, 35%), Equity & Bias (63, 26%), and Privacy & Data (59, 25%). Most policies are coded as medium or low impact (218, 91%), with only 22 (9%) coded as high impact (Fig. 1B). Taken together, these distributions indicate that health-AI activity is still largely governed by transparency-oriented and advisory measures, such as guidance, commissions, and voluntary standards, rather than by prescriptive rules that directly specify when and how clinicians may use AI tools.

Patterns also differ across modules. State policies span the full range of impact ratings, from low-impact resolutions and study commissions to higher-impact statutes and executive actions. Federal, sector-specific, and international modules likewise include policies in all three impact categories, whereas voluntary standards in the snapshot are all medium or low impact, consistent with their role as soft-law instruments.

To see how the highest-impact policies operate, we examined their primary governance mechanisms. Among the 22 high-impact policies, nearly half (10/22; 45%) primarily set substantive use conditions and restrictions on when and how AI can be deployed in health care. The remainder are split between policies that focus on organizational governance and processes (4/22; 18%), information, documentation, and disclosure duties (3/22; 14%), and equity, safety, and assurance regimes (3/22; 14%), with the smallest group centered on strategy, capacity-building, and exploratory actions (2/22; 9%) (Supplementary Table 2). These mechanisms are unevenly distributed across modules: 9 of 10 high-impact instruments that set substantive use conditions and restrictions are state policies, equity, safety, and assurance regimes appear predominantly in sector-specific and international instruments, and strategy and capacity-building actions are concentrated in federal and international measures. Taken together, this distribution shows that even the highest-impact instruments mostly structure governance, documentation, and assurance around AI use rather than imposing blunt bans or permissions.

### Surge and diffusion in 2024–2025

Annual counts of policies remain low and relatively flat in the early years, then rise steadily and peak in 2024–2025, when most policies in the snapshot are issued (Fig. 1C). A similar pattern appears for unique issuing bodies (Fig. 1D; Supplementary Table 1). The number of issuers rises from single digits early in the series to several dozen by 2024–2025, with more than one hundred contributors, indicating that health-AI governance is diffusing across jurisdictions and institutional settings rather than remaining with a few specialist regulators.

### Equity, safety, and stakeholder patterns in governance

Many policies carry more than one keyword tag, and co-tagging patterns underscore that health-AI instruments typically address several concerns rather than a single issue. Among policies tagged for Equity & Bias (*n* = 63), 50 (79%) also carry a Transparency & Governance tag and 32 (51%) carry a Clinical Quality & Efficacy tag. Among policies tagged for Safety and Risk (*n* = 114), 98 (86%) also carry Transparency & Governance and 58 (51%) carry Clinical Quality & Efficacy (Supplementary Table 3). These overlaps indicate that equity and safety concerns are usually embedded in broader transparency and performance requirements instead of being addressed in isolation.

Stakeholder tag distributions reveal a similar concentration of attention. Providers & Health Systems, Regulators & Government, and Developers & Vendors are the most frequently tagged audiences, appearing on 175 (73%), 148 (62%), and 138 (58%) policies, respectively, whereas Patients & Public (128 policies, 53%) and Payers & Purchasers (63 policies, 26%) appear less often (Supplementary Table 4). This pattern suggests that many policies are framed primarily around implementers and overseers rather than patients or payers.

## Discussion

The January 1, 2026, HAPI snapshot suggests that health-AI governance has expanded rapidly in recent years, remains dominated by transparency-oriented, advisory instruments rather than binding clinical rules, and has diffused across more than one hundred issuing bodies. Equity and safety concerns are usually addressed within broader governance and performance requirements, and most concrete obligations fall on providers and health systems, regulators, and developers rather than patients or payers. Taken together, these patterns suggest a governance environment where expectations about documentation, oversight, and risk management accumulate faster than clear, enforceable rules for specific AI uses, creating challenges for health systems, developers, and policymakers interpreting overlapping signals while planning deployments.

Several related features of the current landscape emerge from these analyses. First, governance-mechanism coding for the 22 high-impact policies suggests that many of these instruments work by setting requirements for how AI systems are developed, deployed, and overseen. These include rules about when tools can be used, what monitoring is needed, what documentation must be kept, and what internal review processes organizations should have, rather than broad new individual rights or simple bans on AI use. Second, co-tagging patterns suggest that equity- and safety-oriented measures often appear alongside broader governance and performance requirements rather than on their own. Tags for Equity & Bias or Safety & Risk usually appear alongside Transparency & Governance and Clinical Quality & Efficacy, which suggests that fairness and risk are addressed through requirements for documentation, monitoring, and performance management inside broader governance measures rather than through standalone equity or safety policies. Third, stakeholder tags show that most concrete requirements in these policies are directed at providers and health systems, regulators, and developers, whereas patients and payers are less often described as the main group expected to act. Taken together, these patterns suggest that emerging health-AI governance often works by shaping how organizations design, test, and oversee AI systems, rather than by directly regulating how patients experience or control the use of these tools.

These patterns suggest different roles for various institutions in shaping how AI tools are governed in health care. In this snapshot, state statutes make up most of the high-impact policies coded as setting substantive conditions and restrictions on whether and under what circumstances certain tools can be used. Sector-specific regulators and international bodies appear more often in policies focused on documentation, governance, and assurance practices that health systems and vendors are expected to build around those tools once they are in use. Two medium-impact instruments illustrate how transparency- and governance-oriented measures shape expectations without directly authorizing or prohibiting systems. California’s AB-489^3^ emphasizes disclosures about AI use in health care rather than prescribing specific tools, and the NIST Artificial Intelligence Risk Management Framework^4^ sets expectations for documentation, risk assessment, and governance as a voluntary, cross-sector standard relevant to health-AI deployments. Both are coded as medium impact in HAPI; they do not themselves authorize or prohibit clinical AI systems but help define responsible deployment and due diligence in a fragmented policy environment.

**Fig. 2:**
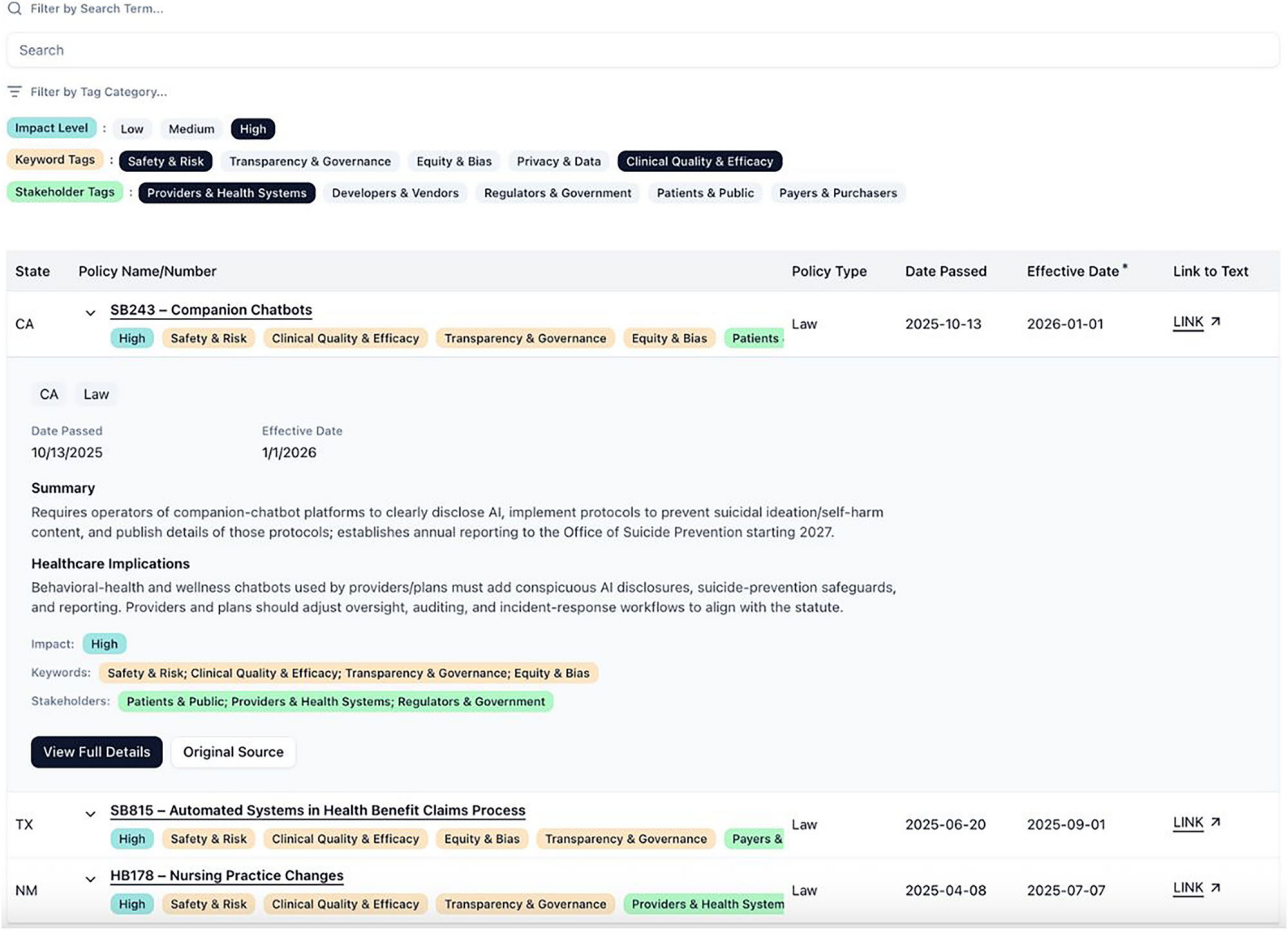
Health & AI Policy Index (HAPI) web interface. Screenshot of the State policies table filtered to high-impact laws, with California SB243 – Companion Chatbots expanded in place. The expanded view shows structured metadata (jurisdiction, policy type, dates, status, impact), keyword and stakeholder tags, and brief “Summary” and “Healthcare implications” fields, along with a link to the source text; additional rows below illustrate the consistent tabular layout across policies.

Against this patchwork, HAPI is intended as infrastructure for navigating and making sense of fragmented rules. Health systems can filter by state, stakeholder tags, and themes such as Safety & Risk or Clinical Quality & Efficacy to see which instruments are likely to affect local governance and deployments and whether their state already has high-impact statutes or regulations. For example, a hospital compliance lead deciding whether to deploy a new prediction model can filter the state-policies table to their state and the Safety & Risk and Providers & Health Systems tags, then expand rows to review summaries and follow links to legal text before bringing options to a governance committee. Developers and vendors can focus on entries tagged for developers and vendors and regulators and government to map the main agencies and standards shaping compliance, while policymakers can benchmark draft proposals against how peer jurisdictions address similar issues across modules. Each module is presented as a single table with sticky headers and sortable columns, and expanding a row reveals structured metadata, brief “Summary” and “Healthcare implications” fields, and a link to the source text (Fig. 2). This layout supports quick scanning and sharing with clinical, legal, and operational teams, reducing the need to navigate multiple pages.

HAPI sits alongside, rather than replaces, other policy-tracking tools and resources. AI-law maps, health-AI atlases, and global AI strategy trackers provide important coverage of statutory activity and high-level regulatory developments, while legal databases and counsel remain essential for authoritative interpretation. HAPI’s main contribution is a health-specific, curated lens: it filters for AI and health relevance, applies tags for sorting, highlights trends, and presents information in a simple interface, offering a structured backbone that other tools, users, and generative AI systems can build on.

### Limitations

This analysis reflects a single snapshot of a rapidly evolving landscape. Policies issued or revised after January 1, 2026, are not captured. Coverage is selective: HAPI focuses on formal written instruments and widely cited standards and does not systematically include enforcement actions, payer decisions, internal institutional policies, or many non-U.S. jurisdictions. We coded primary governance mechanisms only for the small subset of high-impact policies and did not code a single primary purpose or mechanism for every policy in the corpus, so deeper analyses of how specific policy moves vary across modules and issuers remain for future work.

Tag assignments and impact ratings are judgment-based. Although we use written definitions, routine quality checks, and a basic inter-rater check for impact ratings, agreement was modest, and some misclassification is possible, especially for borderline and cross-cutting policies.

Sustainability is another practical constraint, because continued coverage and updates currently depend on institutional support rather than dedicated funding or a broader volunteer community. Exploring a more open, community-driven model is a potential future direction.

Finally, the analysis is descriptive. Counts and patterns across tags, impact levels, and issuers can highlight where activity is concentrated and how it is changing, but they cannot substitute for jurisdiction-specific legal analysis or systematic evaluation of how individual AI systems perform and are governed in practice.

## Conclusion

We analyzed 240 policies in the HAPI snapshot to describe how health-AI governance is taking shape across governments, sector regulators, international bodies, and standards organizations. The landscape appears to have grown rapidly, with activity diffusing across many issuers, remaining weighted toward transparency-oriented advisory instruments rather than binding clinical rules, and often addressing equity and safety through broader requirements on providers, regulators, and developers. By organizing policies into a health-focused registry with consistent metadata, thematic and stakeholder tags, and simple impact classifications, HAPI helps health systems, developers, and policymakers see how diverse measures fit together, identify which instruments are likely to matter most, and recognize gaps that may warrant action. As AI becomes embedded in health care, resources like HAPI can help turn scattered policies into a clearer foundation for governing how these tools are designed, deployed, and overseen.

## Supplementary information

Supplementary Information V3

Supplementary_Data_1_HAPI_snapshot Submission 3

## Data Availability

All data used in this Perspective are drawn from the Health & AI Policy Index (HAPI), a public registry of AI policies affecting health care. The analytic dataset underlying this article includes 240 policies and is frozen as of January 1, 2026. A versioned export of this snapshot, including the policy-level variables analyzed here, is provided as Supplementary Data 1, with additional aggregated tabulations in Supplementary Tables 1 through 4 of the Supplementary Information. The public HAPI website is updated weekly, so later versions of the index may contain additional policies or revisions that are not part of this analytic snapshot.
