# Supplementary material for: Mapping AI regulation in health care with the Health & AI Policy Index
Source: NPJ Digit Med. 2026 May 25;9:413. doi: 10.1038/s41746-026-02734-y (PMC13226646; doi:10.1038/s41746-026-02734-y)
Supplement: Supplementary file 1 — Supplementary Information V3 [file 41746_2026_2734_MOESM1_ESM.pdf]

Table of Contents

Supplementary Note 1. Illustrative examples for tag and impact assignments.

- Keyword tag families: illustrative examples.
- Impact levels: illustrative examples.
- Summary of inter-rater reliability for impact ratings.

Supplementary Table 1. Policies and issuing bodies by year, 2019–2025.

Supplementary Data 1. HAPI analytic snapshot (January 1, 2026).

Supplementary Table 2. Primary governance mechanisms among high-impact policies in the Health & AI Policy Index (HAPI) analytic snapshot.

Supplementary Table 3. Co-occurrence of Equity & Bias and Safety & Risk keyword tags in the January 1, 2026 HAPI snapshot.

Supplementary Table 4. Frequency of stakeholder tags in the January 1, 2026 HAPI snapshot.

Supplementary Note 1. Illustrative examples for tag and impact assignments.

This note provides additional examples of how the keyword tag families and impact levels defined in the main Methods section were applied in borderline cases, and briefly summarizes the inter-rater reliability check for impact ratings.

1. Keyword tag families: illustrative examples.

Example 1 – Equity language without concrete requirements.

A state statute requires developers of AI-enabled decision support tools used in health care to document training data, evaluation methods, and limitations, and to provide that documentation to institutional customers. Toward the end of the statute, there is a short clause encouraging attention to “potential disparate impacts” but no specific bias testing or reporting requirements.

Assigned tags: Transparency & Governance.
Not assigned: Equity & Bias.

Rationale: The main operative provisions concern documentation, disclosure, and institutional governance. Equity is mentioned only at a high level, without concrete obligations to test for or report bias, so an Equity & Bias tag is not added. Under this approach, general references to fairness or equity that lack specific requirements do not by themselves trigger an Equity & Bias tag.

Example 2 – Concrete bias testing requirements.

An international framework on ethical AI includes a section requiring organizations to “identify, assess, and mitigate bias” in AI systems, with specific expectations for subgroup performance analysis and documentation of results.

Assigned tags: Equity & Bias; Transparency & Governance.

Rationale: Here, equity and bias are operationalized through explicit testing and monitoring expectations, so an Equity & Bias tag is assigned. Where the same instrument also requires documentation or reporting of these activities, a Transparency & Governance tag is added as well.

2. Impact levels: illustrative examples.

Impact levels reflect a judgment about the expected strength and immediacy of a policy's influence on health-AI governance, based on the type of instrument, its scope, and how likely it is to shape decisions by health-care organizations, payers, or vendors.

Example 3 – Binding law with clear obligations.

A state statute prohibits certain uses of AI chatbots in direct patient communications unless specific safeguards are met, and assigns enforcement authority to the state health department with explicit penalties for violations.

Assigned impact: High.

Rationale: The statute creates new, binding obligations with clear enforcement mechanisms that directly affect how health-care organizations deploy AI in patient-facing contexts. It is therefore classified as high impact.

Example 4 – Detailed nonbinding guidance versus broad strategy.

A federal health agency issues detailed guidance on validation, documentation, and monitoring practices for AI-enabled clinical decision support tools. The guidance is formally nonbinding but is widely cited in regulatory submissions and procurement language. A separate national AI strategy document describes goals for using AI in health care and calls for future development of regulations and standards, but does not itself create new obligations.

Assigned impact: Medium for the detailed guidance; Low for the national AI strategy.

Rationale: The detailed guidance is specific and likely to shape expectations for developers and health systems even without formal enforceability, so it is classified as medium impact. The broad strategy document signals priorities but has limited direct operational consequences in the near term, so it is classified as low impact.

3. Inter-rater reliability for impact levels.

Impact levels were initially assigned by the HAPI editor. To provide a basic check on consistency, two independent reviewers from our department with expertise in AI in health care coded impact levels (high, medium, low) for a 10 percent sample of policies (n = 24) drawn across modules, blinded to the original codes. Pairwise agreement with the editor's ratings was 58 percent and 63 percent. Discrepant cases were reviewed together and used to clarify wording and borderline examples in the impact descriptions; the final impact ratings in the analytic dataset reflect the reconciled codes.

Supplementary Table 1. Number of policies and unique issuing bodies added per year, 2019–2025.

Counts reflect the 240 policies in the January 1, 2026 analytic snapshot of HAPI across all five modules (U.S. state policies, U.S. federal policies, sector-specific regulation and guidance, international frameworks, and voluntary standards). “Policies added” counts policies first included in HAPI in a given year. “Unique issuing bodies added” counts organizations that appear in HAPI for the first time in that year, including state legislatures and executives, U.S. federal agencies, international organizations, and standards bodies; these counts therefore are not limited to state-level entities.

Year	Policies Added	Unique Issuing Bodies Added
2019	3	3
2020	4	2
2021	13	8
2022	14	11
2023	24	20
2024	67	42
2025	111	73

Source: Health & AI Policy Index (HAPI), snapshot frozen January 1, 2026.

Supplementary Data 1. HAPI analytic snapshot (January 1, 2026).

Excel file with one row per policy, policy name, module, policy type, issuing body, key dates, impact level, keyword tags, stakeholder tags, and links to source text.

Supplementary Table 2. Primary governance mechanisms among high-impact policies in the Health & AI Policy Index (HAPI) analytic snapshot (n = 22).

Each high-impact policy was assigned a single primary governance mechanism: substantive use conditions and restrictions; organizational governance and processes; information, documentation, and disclosure duties; equity, safety, and assurance regimes; or strategy, capacity-building, and exploratory actions. Counts and percentages refer to the share of all high-impact policies in the analytic dataset; the final column indicates which HAPI modules most commonly contain each mechanism type.

Primary mechanism (high-impact policies only)	No. of policies	Percent of high-impact policies	Dominant modules (no.)
Substantive use conditions & restrictions	10	45	State (9), International (1)
Organizational governance & processes	4	18	State (2), Federal (1), International (1)
Information, documentation, and disclosure duties	3	14	State (3)
Equity, safety, and assurance regimes	3	14	Sector-Specific (2), International (1)
Strategy, capacity-building, and exploratory actions	2	9	Federal (1), International (1)
**Total**	22	100	

Supplementary Table 3. Co-occurrence of Equity & Bias and Safety & Risk keyword tags in the January 1, 2026 HAPI snapshot.

Number of policies tagged for Equity & Bias and Safety & Risk and the frequency with which these tags co-occur with Transparency & Governance, Clinical Quality & Efficacy, and each other. Because policies can carry multiple keyword tags, counts across columns do not sum to the total number of policies.

Base tag	Policies with base tag, n	…also tagged Transparency & Governance, n	…also tagged Clinical Quality & Efficacy, n
Equity & Bias	63	50	32
Safety & Risk	114	98	58

Supplementary Table 4. Frequency of stakeholder tags in the January 1, 2026 HAPI snapshot.

Number and percentage of policies carrying each stakeholder tag (Providers & Health Systems, Developers & Vendors, Regulators & Government, Patients & Public, and Payers & Purchasers). Percentages use the total number of policies in the snapshot (n = 240) as the denominator; policies can carry multiple stakeholder tags, so percentages sum to more than 100 percent.

Stakeholder group	Policies with this stakeholder tag, n	Policies with this stakeholder tag, % of all policies*
Providers & Health Systems	175	73
Developers & Vendors	138	58
Regulators & Government	148	62
Patients & Public	128	53
Payers & Purchasers	63	26
